# Candidate gene association study suggests potential role of dopamine beta-hydroxylase in pain heterogeneity in sickle cell disease

**DOI:** 10.3389/fgene.2023.1193603

**Published:** 2023-06-13

**Authors:** Nilanjana Sadhu, Ying He, Yingwei Yao, Diana J. Wilkie, Robert E. Molokie, Zaijie Jim Wang

**Affiliations:** ^1^ Department of Pharmaceutical Sciences, University of Illinois Chicago College of Pharmacy, Chicago, IL, United States; ^2^ Comprehensive Sickle Cell Center, University of Illinois Chicago, Chicago, IL, United States; ^3^ Department of Biobehavioral Nursing Science, University of Florida College of Nursing, Gainesville, FL, United States; ^4^ Jesse Brown Veteran’s Administration Medical Center, Chicago, IL, United States; ^5^ Division of Hematology/Oncology, University of Illinois Chicago College of Medicine, Chicago, IL, United States; ^6^ Department of Neurology and Rehabilitation, University of Illinois Chicago College of Medicine, Chicago, IL, United States; ^7^ Department of Biomedical Engineering, University of Illinois Chicago College of Engineering, Chicago, IL, United States

**Keywords:** pain, sickle cell disease, chronic pain, acute pain, pharmacogenenomics and personalised medicine

## Abstract

**Introduction:** Pain is a lifelong companion of individuals with sickle cell disease (SCD) and has a severe impact on their quality of life. Both acute crisis pain and chronic non-crisis pain exhibit high variability between individuals, making it difficult to effectively manage sickle cell-related pain. We investigated the role of dopamine beta-hydroxylase (DBH) gene polymorphisms on pain variability in SCD. DBH is a key enzyme in the catecholamine biosynthesis pathway that catalyzes the conversion of dopamine to norepinephrine, both of which are known mediators of pain and pain-related behaviors.

**Methods:** Acute crisis pain-related utilization and non-crisis chronic pain scores of 131 African Americans with SCD were obtained.

**Results and discussion:** Association analyses revealed that the T allele of upstream variant rs1611115 and downstream variant rs129882 correlated with higher severity of chronic pain in an additive model. On the other hand, the A allele of missense variant rs5324 associated with lower risk of both acute crisis pain and chronic pain. Similarly, the C allele of intronic variant rs2797849 was associated with lower incidence of acute crisis pain in the additive model. In addition, tissue-specific eQTL revealed that the T allele of rs1611115 correlated with decreased expression of *DBH* in the frontal cortex and anterior cingulate cortex (GTEx), and decreased expression of *DBH-AS1* in blood (eQTLGen). Bioinformatic approaches predicted that rs1611115 may be altering a transcription factor binding site, thereby, contributing to its potential effect. Taken together, findings from this study suggest that potential functional polymorphisms of *DBH* may modulate pain perception in SCD.

## Introduction

The dopamine beta-hydroxylase (DBH) is a catalytic enzyme found in secretory vesicles of noradrenaline producing neurons in both the central and peripheral nervous systems (Cubells et al., 1998). It catalyzes the hydroxylation of dopamine to produce norepinephrine in these neurons (Diliberto and Allen, 1981) and is secreted along with the neurotransmitters during synaptic transmission (Weinshilboum et al., 1971). Therefore, DBH can be detected in the cerebrospinal fluid (CSF) and plasma, and polymorphisms in *DBH* have been found to alter plasma and CSF levels of the protein (Tunbridge et al., 2019). Altered DBH expression or activity would result in an imbalance of dopamine and norepinephrine. Since both of these neurotransmitters are key modulators in pain signaling (Jasmin et al., 2002; Leventhal et al., 2007; Jarcho et al., 2012), it was hypothesized that polymorphisms in *DBH* may be associated with variability in pain perception.

In that regard, we investigated the potential role of DBH variants on the heterogeneity of acute and chronic pain phenotypes in sickle cell disease (SCD). SCD is a debilitating genetic blood disorder in which patients suffer from pain throughout their lives, starting as early as 1 year after birth (Platt et al., 1991; Miller et al., 2000). Acute pain is caused by frequent and recurring episodes of ischemia-like events where deformed red blood cells agglomerate and block microvasculature in different organs (Manwani and Frenette, 2013). These events are known as painful vaso-occlusive crisis and are a leading cause for hospitalization among SCD patients (Shah et al., 2019). Chronic pain, on the other hand, is persistent daily pain experienced even on non-crisis days and believed to be caused by complex neuropathic pain mechanisms (Wang et al., 2010; He et al., 2016). It has been found that both pain phenotypes exhibit high variability between patients, thus rendering the assessment and management of pain in SCD a challenging task (Okpala and Tawil, 2002; Wilkie et al., 2010). A better understanding of the various genetic factors that modulate sickle cell pain variability can help us devise more effective pain management strategies.

Previously, we reported that genetic variants of other enzymes in the catecholamine biosynthesis pathway such as *COMT*, *GCH1*, *PNMT* can contribute to the inter-individual variability of pain in SCD (Jhun et al., 2014; Sadhu et al., 2018; Sadhu et al., 2020). In this study, we evaluate the role of genetic variants in another key monoaminergic enzyme DBH. The *DBH* gene is located on chromosome 9 and consists of 12 exons. Five single nucleotide polymorphisms (SNPs) rs1611115, rs2797849, rs5320, rs5324, and rs129882 spanning the gene were assessed in this study. rs1611115 is located upstream of the gene near its proximal promoter region. rs2797849 is an intronic SNP, whereas rs5320 and rs5324 are non-synonymous variants lying in exon 3 and 4, respectively. Finally, rs129882 is located in the 3’ untranslated region of *DBH*.

## Methods

### Study design

This study protocol was approved by the University of Illinois (UI) at Chicago’s Institutional Review Board. SCD patients were recruited at the Sickle Cell Clinic of the UI Hospital and Health Sciences System. Written informed consent of adult participants (aged 18 or older), and child assent and parental consent for participants under the age of 18 were obtained during subject recruitment. Eligibility criteria of subjects are provided in greater detail in previous publications (Ezenwa et al., 2014; Dyal et al., 2020). All subjects included in this study (N = 131) identified themselves as African Americans. Of the 131 subjects, 45 were male and 86 were female, their ages ranging from 15 to 70 years. A majority of the subjects (N = 102) had SS type sickle cell disease, i.e., homozygous for hemoglobin S. Subject demographics are outlined in Table 1.

**TABLE 1:** Subject demographics.

Age	Range = 15–70 years
	Mean = 34.3 ± 11.8 years
Sex	Male, N = 45
	Female, N = 86
Sickle cell type^a^	HbSS, N = 102
	HbSC, N = 15
	Others, N = 14

^a^ Sickle cell type: HbSS (homozygous with HbS), HbSC (co-inherited HbS and HbC), others include SCD-sickle β^+^ thalassemia, SCD-sickle β^0^ thalassemia and SCD-sickle *α* thalassemia.

### Assessment of pain

Two types of pain phenotype data were collected—acute crisis pain and chronic non-crisis pain. Annual hospitalization counts of subjects due to crisis pain, referred to as utilization, was used as a marker for acute crisis pain measurement. Utilization data of patients were obtained mainly from the UI medical records of emergency department and acute care center visits (Ezenwa et al., 2014). Biweekly telephone calls to patients were made to record any out-of-UI-system visits. During their regular clinic visits, patients were asked to score various aspects of their baseline pain experience on a digital tool adapted from the McGill Pain Questionnaire. Scores from each category of questions, i.e., pain sites, pain intensity, pain pattern and quality, were aggregated and scaled to a range of 0–100 as described in earlier studies (Wilkie et al., 2010; Wilkie et al., 2015). This was reported as the Composite Pain Index (CPI) and used as a marker for chronic non-crisis pain measurement.

### DNA isolation and genotyping

Blood or buccal swab samples were collected from subjects for genotyping. DNA was isolated from buccal swab samples using a modified phenol-chloroform method (Vandenbergh et al., 2003). DNA isolation from blood samples was performed using a modified salting-out procedure (Miller et al., 1988) or with the QuickGene DNA whole blood extraction kit (AutoGen, Holliston, MA). Candidate SNP genotyping was performed at the UIC Research Resource Center using the Agena Bioscience MassARRAY^®^ System. SNPs included in this study had a call rate of at least 90% and were in Hardy Weinberg equilibrium (*p* > 0.05).

### Statistical analysis

Based on the distribution of the pain phenotype data in our study sample, multiple linear regression was used to analyze CPI scores, whereas negative binomial regression was used to model utilization scores. Additive, and dominant genetic models of penetrance were used to assess the effect of the minor variant allele as compared to that of the major ancestral allele. All regression models were adjusted for covariates age, sex, and sickle type. R package ‘MASS’ was utilized for regression analysis.

Expression quantitative loci (eQTL) data were obtained from Genotype-Tissue Expression (GTEx) (version November 2019) for SNPs rs1611115 (chr9_133635393_T_C_b38), rs2797849 (chr9_133636819_G_C_b38), rs5320 (chr9_133642351_G_A_b38), and rs129882 (chr9_133658547_C_T_b38). The minor allele of rs5324 (chr9_133643536_G_A_b38) being less than 1% in occurrence was not included in the GTEx database. *DBH* gene expression in 11 different brain tissue types (preserved as fresh frozen tissue and whose main sampling site was at the Miami Brain Bank) were assessed. The tissue types included Amygdala, Anterior Cingulate Cortex, Caudate (basal ganglia), Cerebellar Hemisphere, Frontal Cortex, Hippocampus, Hypothalamus, Nucleus accumbens (basal ganglia), Putamen (basal ganglia), Spinal cord (cervical c-1), and Substantia nigra. Violin plots of significant associations between SNPs and tissue-specific gene expression levels were downloaded from the GTEx portal. We also looked up cis-eQTLs in the eQTLGen Consortium database (Võsa et al., 2021). Finally, functional analyses of rs1611115 was performed using web-based bioinformatic tools PROMO (http://alggen.lsi.upc.es/) and SNPinfo: FuncPred (https://snpinfo.niehs.nih.gov/), to predict putative transcription factor binding sites that may be affected by the SNP (Messeguer et al., 2002; Farré et al., 2003; Xu and Taylor, 2009).

## Results

Acute and chronic pain phenotype data of study subjects have been summarized in Table 2. The heterogeneity of both pain phenotypes is reflected in the wide range of pain scores of the sample population. The 12-month utilization rate ranged from zero to 38, with a median of three utilizations per year. CPI scores on a scale of zero to 100 ranged from 14.8 to 86.5, with a mean score of 40.6. Neither utilization rate, nor CPI were found to be significantly different between males and females. Similarly, no significant differences in the pain phenotypes were observed based on sickle cell type. The distribution of the major and minor alleles and the corresponding genotypes in the study population is provided in Table 3. Comparison of allele frequencies with the African ancestry in Southwest US (ASW) population from the 1,000 genomes project showed that rs1611115, rs5320, rs5324, and rs129882 had comparable major allele frequencies. The major allele G of rs2797849 was, however, found to be more frequent in this study cohort (74%) than that in the ASW cohort (63%).

**TABLE 2:** Summary of pain phenotype data.

	Overall	Male	Female	P-value (Male vs Female)	HbSS	HbSC + Others	P-value (HbSS vs HbSC + Others)
Utilization, Median (Range)	3 (0 – 38)	2 (0 – 23)	3 (0 – 38)	.12	3 (0 – 23)	3 (0 – 38)	.90
Composite Pain Index (CPI) Mean ± SD	40.6 ± 13.4	38.1 ± 13.6	41.9 ± 13.2	.13	40.1 ± 13.7	42.4 ± 12.5	.39

HbSS (homozygous for HbS), HbSC (co-inherited HbS and HbC), Others include SCD-sickle β+ thalassemia, and SCD-sickle β0 thalassemia.

P-values from (i) unpaired two-sample Wilcoxon rank-sum test (two-sided) for Utilization, and (ii) unpaired two-sample t-test (two-sided) for Composite Pain Index are reported for each comparison.

**TABLE 3:** *DBH* polymorphism genotype and allele frequencies.

dbSNP ID (Chr:BP^a^)	ASW allele (MAF)^b^	Allele	N (%)	Genotype	N (%)
rs1611115 (9:133635393)	C (0.795)	C	206 (83)	CC	87 (70)
		T	42 (17)	CT	32 (26)
				TT	5 (4)
rs2797849 (9:133636819)	G (0.631)	G	189 (74)	GG	73 (57)
		C	67 (26)	GC	43 (34)
				CC	12 (9)
rs5320 (9:133642351)	G (0.877)	G	224 (85)	GG	95 (73)
		A	38 (15)	GA	34 (26)
				AA	2 (2)
rs5324 (9:133643536)	G (0.910)	G	229 (94)	GG	107 (88)
		A	15 (6)	GA	15 (12)
				AA	0 (0)
rs129882 (9:133658547)	C (0.762)	C	212 (74)	CC	87 (67)
		T	46 (26)	CT	38 (29)
				TT	4 (3)

^a^ Chromosomal location from GRCh38.

^b^ ASW is African ancestry in Southwest US population in the 1,000 genomes project, MAF is the major allele frequency.

Multilinear regression models evaluating the association between these SNPs and CPI scores revealed that the minor alleles of rs1611115, rs5324, and rs129882 significantly associated with chronic pain (Table 4). The T allele of rs1611115 was found to significantly associate with higher CPI scores in the additive model (B = 4.74, *p* = 0.032). Similarly, the T allele of rs129882 also associated significantly with higher CPI scores (Additive: B = 4.74, *p* = 0.034; Dominant: B = 6.86, *p* = 0.008). Whereas the minor allele A of rs5324 associated with lower CPI scores (B = −9.59, *p* = 0.010). Since there were no subjects with homozygous recessive genotype for the SNP rs5324, the additive and dominant models were identical. As seen in Table 5, the rs5324 A allele associated significantly with decreased utilization rate of acute crisis pain (IRR = 0.54, *p* = 0.035) as well. The C allele of rs2797849 too was found to associate with decreased utilization scores (IRR = 0.75, *p* = 0.042).

**TABLE 4:** Association analyses of Composite Pain Index with *DBH* polymorphisms.

SNP	Model	B^a^ (97.5% CI)	*p*-value
rs1611115	Additive (CC vs. CT vs. TT)	4.74 (0.42, 9.06)	0.032^b^
	Dominant (CC vs. CT + TT)	3.00 (−2.28, 8.27)	0.263
rs2797849	Additive (GG vs. GC vs. CC)	−2.95 (−6.59, 0.68)	0.111
	Dominant (GG vs. GC + CC)	−4.72 (−9.51, 0.07)	0.053
rs5320	Additive (GG vs. GA vs. AA)	−0.27 (−5.28, 4.73)	0.914
	Dominant (GG vs. GA + AA)	−0.34 (−5.82, 5.13)	0.901
rs5324	Additive (GG vs. GA vs. AA)	−9.59 (−16.86, −2.31)	0.010^b^
	Dominant (GG vs. GA + AA)	−9.59 (−16.86, −2.31)	0.010^b^
rs129882	Additive (CC vs. CT vs. TT)	4.74 (0.37, 9.12)	0.034^b^
	Dominant (CC vs. CT + TT)	6.86 (1.84, 11.87)	0.008^b^

^a^ Unstandardized regression coefficient.

^b^ *p*-value < 0.05.

**TABLE 5:** Association analyses of utilization scores with *DBH* polymorphisms.

SNP	Model	IRR (95% CI)^a^	*p*-value
rs1611115	Additive (CC vs. CT vs. TT)	1.34 (0.99, 1.85)	0.074
	Dominant (CC vs. CT + TT)	1.32 (0.88, 1.99)	0.172
rs2797849	Additive (GG vs. GC vs. CC)	0.75 (0.57, 0.99)	0.042^b^
	Dominant (GG vs. GC + CC)	0.76 (0.53, 1.09)	0.140
rs5320	Additive (GG vs. GA vs. AA)	0.86 (0.59, 1.26)	0.433
	Dominant (GG vs. GA + AA)	0.85 (0.57, 1.29)	0.439
rs5324	Additive (GG vs. GA vs. AA)	0.54 (0.30, 0.98)	0.035^b^
	Dominant (GG vs. GA + AA)	0.54 (0.30, 0.98)	0.035^b^
rs129882	Additive (CC vs. CT vs. TT)	0.97 (0.70, 1.36)	0.875
	Dominant (CC vs. CT + TT)	0.93 (0.64, 1.38)	0.732

^a^ Incidence Risk Ratio.

^b^ *p*-value < 0.05.

**FIGURE 1:**
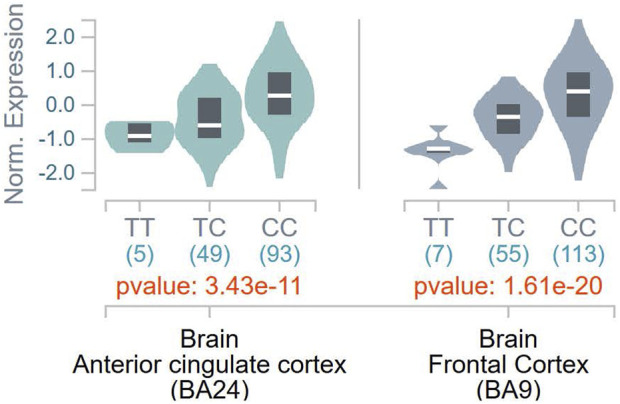
eQTL analysis of *DBH-*rs 1611115 from GTEx database. Violin plots of tissue-specific eQTLs of rs1611115 (chr9_133635393_T_C_b38) for *DBH* (ENSG00000123454.10) expression in Anterior Cingulate Cortex and Frontal Cortex obtained from the GTEx Analysis Release V8 (dbGap Accession phs000424.v8p2). The GTEX Project was supported by the Common Fund of the Office of the Director of the National Institutes of Health and by NCL, NHGRI, NHLBI, NIDA, NIMH, and NINDS.

To ascertain the potential functional role of these SNPs, the GTEx database was queried for tissue-specific association between expression levels of *DBH* and the SNPs. It was found that the T allele of rs1611115 associated with significantly lower expression of *DBH* in the frontal cortex and the anterior cingulate cortex in the GTEx database (Figure 1). Furthermore, rs1611115 was found to be a blood cis-eQTL of *DBH-AS1* in eQTLGen database (*p* = 3.58 × 10^−6^), with the T allele being responsible for decreased gene expression.

**FIGURE 2:**
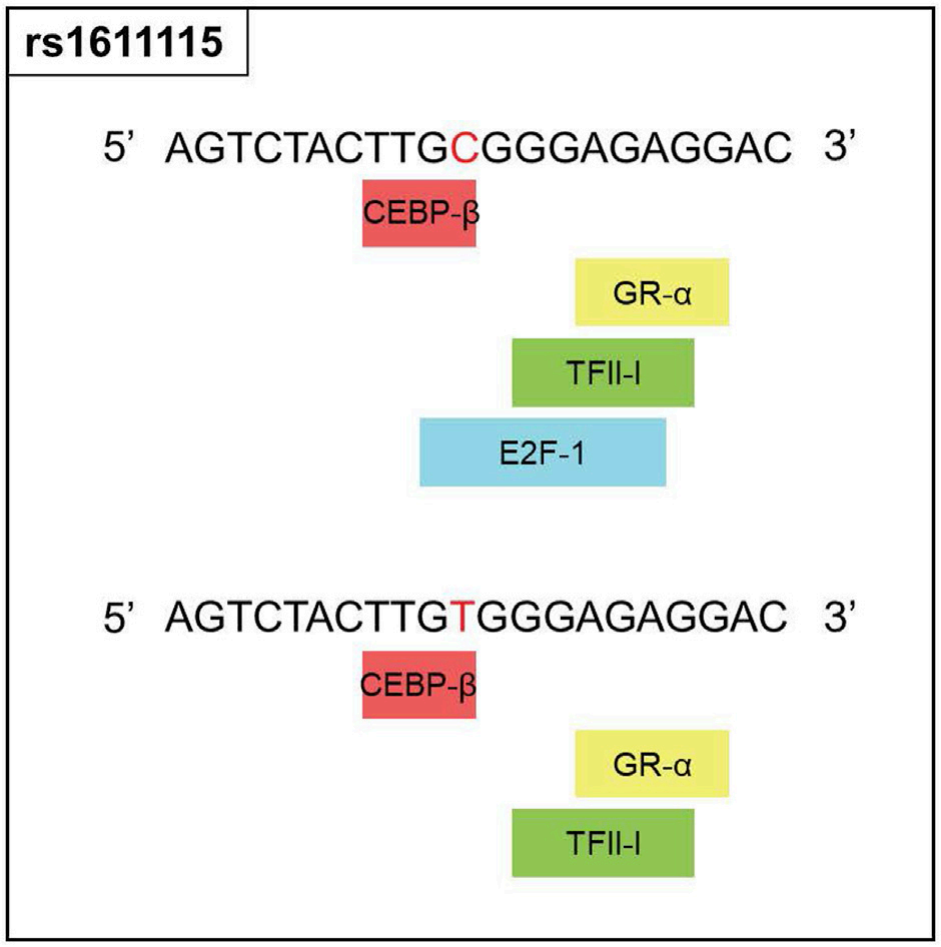
Functional prediction for rs1611115. Changes in the transcription factor blinding sites of rs1611115[C>T] as predicted by PROMO, Polymorphic variation in the input sequence is indicated by red letter. Input sequence was obtained from the dbSNP database.

Since rs1611115 is located near the promoter region of *DBH* and was found to associate with altered expression level of *DBH* in specific regions of the brain, additional functional analysis was performed. Bioinformatic tools PROMO and SNPinfo:FuncPred were used to investigate the potential effect of this polymorphism on transcription factor binding sites (TFBS). It was found that the substitution of the allele C with variant T could lead to the loss of a putative transcription factor binding site, potentially that of E2F1 (Figure 2).

## Discussion

Polymorphisms on the *DBH* gene have been studied for their associations with several neurological disorders such as migraine (Fernandez et al., 2006; Fernandez et al., 2009; Ghosh et al., 2013), Parkinson’s disease (Ghosh et al., 2019), schizophrenia (Cubells et al., 2011; Sun et al., 2018), attention deficit hyperactivity disorder (Kieling et al., 2008), Alzheimer’s disease (Combarros et al., 2010), etc. In this study, we investigated the effect of *DBH* polymorphisms on pain in SCD and found that *DBH* rs5324 associated with both acute crisis pain and chronic pain, with the A allele being pain protective. On the other hand, rs1611115 and rs129882 were associated with chronic pain only, with the T alleles conferring a higher risk of pain. In addition, the C allele of rs2797849 was associated with decreased acute crisis pain.

As noted, rs5324 exhibited association with pain, while the other missense variant rs5320 did not associate with either of the pain phenotypes. In agreement with this observation, a previous study that developed an in-silico protein model of *DBH* predicted that the SNP rs5320 (Ala211Thr) may not alter enzyme activity since it did not lie close to the active site of the enzyme or its tetramer interface (Kapoor et al., 2011). The study also suggested that rs5324 (Asp290Asn) was situated close to the tetramer interface and could potentially interfere with tetramerization of the subunits into the enzyme’s quaternary structure. Thus, the exon SNP rs5324 may alter the enzymatic activity of DBH. It would be interesting to confirm such a hypothesis in future experiments.

Prior research examining the relationship between *DBH* variants and DBH levels in the plasma of African American found that while a few SNPs associated with altered plasma levels of DBH, rs1611115 accounted for the largest variation (30%–50%). The rs1611115 was also found to be in LD with other genetic variants and the T allele associated with significantly lower levels of DBH (Zabetian et al., 2001; Tang et al., 2007; Mustapic et al., 2014). Similarly, rs1611115 has been found to alter DBH proteins levels in the CSF (Weinshilboum et al., 1971). It has also been shown that the effect of *DBH* variants such as rs1611115 on gene expression can be tissue-specific (Barrie et al., 2014). With these studies in sight, the association of *DBH* variants (including rs1611115) with gene expression levels of *DBH* in different brain tissues were *explored on the GTEx database. It should be noted that a majority of the samples in the GTEx database were obtained from white subjects, with African Americans accounting for approximately 13% of the overall cohort. It has been previously shown that the extent to which rs16*11115 accounts for decreased levels of plasma DBH can vary between European Americans and African Americans (Zabetian et al., 2001). Nonetheless, it was found that the T allele of rs1611115 associated with decreased *DBH* expression levels in the frontal cortex tissue and the anterior cingulate cortex (ACC) tissue. This SNP-associated decrease in *DBH* expression may be explained by the loss of a putative transcriptional binding site for transcription factor E2F1. E2F1 belongs to a family of transcriptional factors that primarily regulate expression of genes involved in cellular proliferation (DeGregori et al., 1997; Wu et al., 2001). It has also been shown to mediate neurogenesis and dopamine-induced neuronal apoptosis (Hou et al., 2001; Cooper-Kuhn et al., 2002). However, the role of E2F1 is not limited to cell proliferation alone; thousands of genes contain the E2F1 binding site in their promoter region (Bieda et al., 2006; Iwanaga et al., 2006). E2F1 may activate or repress gene expression depending on the type of binding proteins it is programmed to recruit at that site (Denechaud et al., 2017).

Since DBH catalyzes the conversion of dopamine to noradrenaline, functional polymorphisms in *DBH* would alter the levels of both the neurotransmitters. Dopamine and norepinephrine can have pronociceptive or antinociceptive effects depending on the brain region, tissue type, as well as their release (Pertovaara, 2013; Taylor et al., 2016; Bravo et al., 2019). In one study, *DBH* knockout mice lacking noradrenaline production were found to develop chronic thermal hyperalgesia and exhibited diminished morphine efficacy (Jasmin et al., 2002). The frontal cortex, specifically the prefrontal cortex receives both dopaminergic and noradrenergic innervations from different regions in the midbrain, and their dysfunction have been implicated in pain conditions (Ong et al., 2019). The ACC is also believed to be critical in modulating pain and pain-related behavior (Fuchs et al., 2014; Bliss et al., 2016). Noxious stimuli have been found to activate the ACC in healthy individuals; ACC activation has also been noted in individuals with chronic pain (Talbot et al., 1991; Apkarian et al., 2005). However, the precise roles of dopaminergic and noradrenergic signaling in pain processing within the frontal cortex and ACC remains to be identified.

In summary, SNPs in the *DBH* gene were found to be associated with acute and chronic pain in SCD. The eQTL data revealed that the promoter region variant rs1611115-T also associated with *DBH* expression in specific regions of the brain known to be involved in pain signaling. Bioinformatic analysis indicated that this variant could be potentially involved in altered transcriptional regulation. Haplotype analysis would have been an interesting addition to this study but was not possible due to the relatively small sample size. While this study is exploratory in nature and limited by its sample size, the findings of this candidate-gene study suggest a possible role of *DBH* on pain variability in SCD. Larger genetic association studies and functional genomics experiments are needed to comprehensively understand how the identified SNPs may be altering *DBH* gene expression, its enzymatic activity, and consequently pain perception.

## Data Availability

The original contributions presented in the study are publicly available. This data can be found here: https://data.mendeley.com/datasets/j9nsx69jyg.
